# Rheometer enabled study of cartilage frequency-dependent properties

**DOI:** 10.1038/s41598-020-77758-9

**Published:** 2020-11-26

**Authors:** Stefano Perni, Polina Prokopovich

**Affiliations:** School of Pharmacy and Pharmaceutical Sciences, Cardiff University, King Edward VII Avenue, Redwood BuildingCardiff, CF10 3NB UK

**Keywords:** Materials science, Soft materials, Rheology

## Abstract

Despite the well-established dependence of cartilage mechanical properties on the frequency of the applied load, most research in the field is carried out in either load-free or constant load conditions because of the complexity of the equipment required for the determination of time-dependent properties. These simpler analyses provide a limited representation of cartilage properties thus greatly reducing the impact of the information gathered hindering the understanding of the mechanisms involved in this tissue replacement, development and pathology. More complex techniques could represent better investigative methods, but their uptake in cartilage research is limited by the highly specialised training required and cost of the equipment. There is, therefore, a clear need for alternative experimental approaches to cartilage testing to be deployed in research and clinical settings using more user-friendly and financial accessible devices. Frequency dependent material properties can be determined through rheometry that is an easy to use requiring a relatively inexpensive device; we present how a commercial rheometer can be adapted to determine the viscoelastic properties of articular cartilage. Frequency-sweep tests were run at various applied normal loads on immature, mature and trypsinased (as model of osteoarthritis) cartilage samples to determine the dynamic shear moduli (G*, G′ G″) of the tissues. Moduli increased with increasing frequency and applied load; mature cartilage had generally the highest moduli and GAG depleted samples the lowest. Hydraulic permeability (K_H_) was estimated from the rheological data and decreased with applied load; GAG depleted cartilage exhibited higher hydraulic permeability than either immature or mature tissues. The rheometer-based methodology developed was validated by the close comparison of the rheometer-obtained cartilage characteristics (G*, G′, G″, K_H_) with results obtained with more complex testing techniques available in literature. Rheometry is relatively simpler and does not require highly capital intensive machinery and staff training is more accessible; thus the use of a rheometer would represent a cost-effective approach for the determination of frequency-dependent properties of cartilage for more comprehensive and impactful results for both healthcare professional and R&D.

## Introduction

Cartilage covers the bone extremities subjected to contact during joint movement and the primal function is to assure that the relative movement of the joint surfaces occurs with the low friction possible^1,2^. Articular cartilage is composed of cells (chondrocytes) distributed in a fluid-filled extracellular matrix (ECM)^3^. Cartilage material properties depend primarily on the properties of the ECM, mainly constituted by collagen II and proteoglycans (highly negatively charged biopolymers). Cartilage structure and composition varies from childhood to adulthood (maturation)^4–6^ but it is also affected by chronical (osteoarthritis (OA))^7–9^ and acute diseases (injuries)^10,11^. During these pathological conditions, the thickness of the cartilage tissue is reduced and the relative movement of the joint surfaces is hindered resulting in pain thus affecting significantly patient life quality^12–14^.

Evaluation of cartilage composition and mechanical properties is essential in many aspects of research such as tissue engineering/regeneration and in elucidating processes such as drugs and therapies efficacy, disease onset and progression along with cell signalling. Many methods are available for the study of the composition of cartilage, for example the dimethyl-methylene blue (DMMB)^15^ and chloramine-T reagent and dimethylaminobenzaldehyde (DMBA)^16^ are used to quantify glucosamineglycan (GAG) and collagen content, respectively; live-dead staining microscopy^17^ and MRI^18^ allow the determination of the cartilage thickness and chondrocytes viability while PCR provides information on genes expression^19,20^. However, it has become more and more evident that cartilage mechanical properties are essential to their functionality and such knowledge is pivotal in understanding not only the physiology and pathology of this tissue but also in the development of effective treatments for the diseases affecting it^20–22^. In any material, the ability to absorb the energy of an impact is due to the loss modulus while the ability to return to the original size is controlled by the elastic modulus. Furthermore, for porous materials immersed in a fluid, the relation between elastic and loss moduli is dependent on the ability of the fluid to flow as described by the hydraulic constant. In this respect, cartilage functionality as shock absorber is directly controlled by all of these properties as this tissues is porous and normally in contact with synovial fluid. A critical aspects of tissue engineering of cartilage is the generation of a replacement tissue with mechanical properties matching those of the natural tissue over the several orders of magnitude of loading rates experienced during daily activities^23^.

Relatively simple testing methods are unable to capture accurately the structural complexity that cartilage exhibits; another consideration is that physiological activities (i.e., walking, running) are transient loading and unloading events occurring in < 1 s; hence, the mechanical response of cartilage should be measured under dynamic cyclic conditions over a range of physiologically relevant frequencies^24^. Complex techniques employed in material science such as confined and unconfined compressions^25–28^ have been applied for the characterisation of cartilage structural properties; however they may not be fully satisfactory as they do not provide the necessary oscillatory behaviour as, in these techniques, the cartilage samples are squeezed but not oscillations are applied. Similarly, continuous load—stress relaxation applied to cartilage does not represent the transitory nature of the mechanical stimuli cartilage tissue are subjected^29^. Atomic force microscopy (AFM) nanoindentations^24,30^ and dynamic mechanical analysis (compression frequency sweep)^31–34^ provides the required oscillatory features; however the complexity of their operations (requiring highly trained operators) and the cost of the equipment are the main downsides of these techniques that have limited their utilisation among cartilage focused research groups or in clinical settings for diagnostic purposes.

This highlights the clear need for the development of techniques that allow the study of frequency-dependent cartilage properties through user-friendly and simple devices. For this purpose, we developed an approach based on the use of a commercial rheometer^35^ for the study of frequency dependent mechanical properties of cartilage thus providing a simpler and more cost-effective platform for the investigation of cartilage properties. Rheometry is a well established technique used in the study of soft materials such as polymers, emulsions and foods. G′ (storage modulus) and G″ (loss modulus) of immature, mature and GAG depleted (mimicking early stage of OA) cartilage, along with the corresponding phase angle (δ) and dynamic modulus G*, were determined at frequencies ranging from 10 Hz to 0.001 Hz, applying normal forces of 5 N, 25 N and 50 N. The results were those expected for poro-elastic materials with a bell shaped profile of phase angle (δ) vs. applied load frequency; the value of applied frequency corresponding to a local maximum in the phase angle (f_peak_) was also used to extract values of cartilage hydraulic permeability (K_H_) at different applied loads. The methodology was validated by positive comparison of the rheometer obtained characteristics with results obtained with more complex testing techniques such as dynamic mechanical analysis (compression frequency sweep) and AFM nanoindentation available in literature.

## Materials and methods

### Cartilage samples

**Figure 1:**
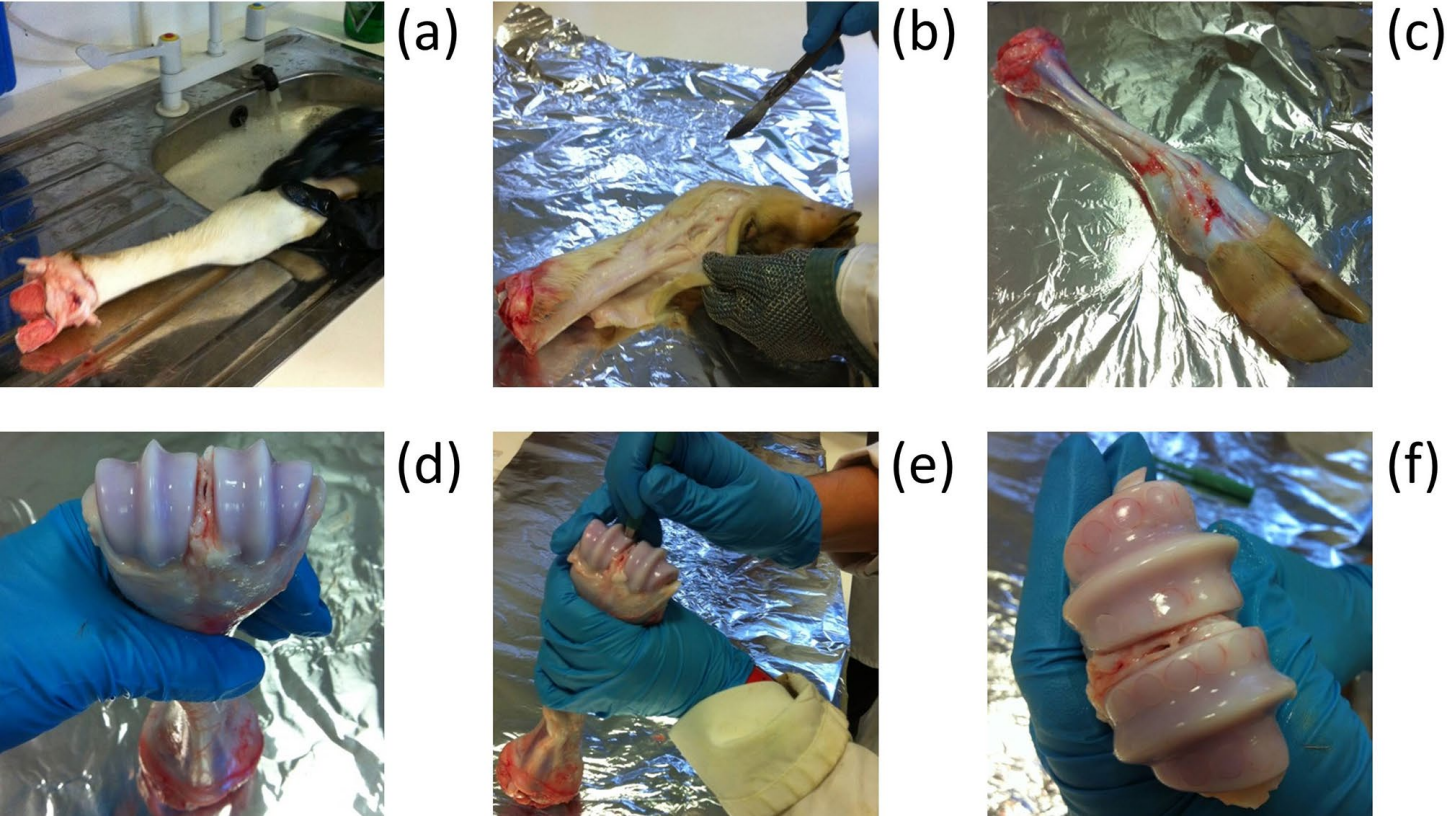
Biopsy process, cleaning legs carefully in order to remove all dirt (**a**); skin removal with a blade gently to not damage the cartilage underneath (**b**); joint opening with a scalpel (**c**, **d**); full depth explants excised using biopsy punches (**e**, **f**).

Bovine steers matured (over 18 months old) and immature (7-day-old) cartilage from bovine steers was obtained from local abattoirs. The age range of the mature donors was between 18 and 28 months. Articular cartilage explants were surgically removed under sterile conditions from metacarpo-phalangeal joints^36^. First, legs were washed in order to remove all dirt present. Then the skin was removed with a blade paying great attention not to damage the cartilage underneath. The ankle joint was then exposed, and full depth explants were excised using a 5 mm diameter biopsy punches (Fig. 1). Explants were washed to remove blood and subchondral bone.

GAG depleted samples were obtained incubating the cartilage samples in a solution of trypsin 1 mg/ml in PBS for 24 h at 37 °C and washed three times in fresh PBS. Trypsin was used to deplete cartilage extracellular matrix of GAG content in order to simulate the ECM osteolysis observed in osteoarthritis^37^, additionally bovine cartilage have been shown to be a good model for human tissues^31^.

Samples were used immediately after explant or GAG depletion without storing.

Explants were weighed wet then incubated for 24 h in papain digestion buffer (20 mm sodium acetate pH 6.8, 1 mm EDTA, 2 mm dithiothreitol and 300 μg/ml papain) at 50 °C.

GAG content was measured with the DMMB assay against standards of chondroitin sulphate of sharks^15^. Hydroxyproline content was determined on acid hydrolysates of papain digested samples^16^.

**Figure 2:**
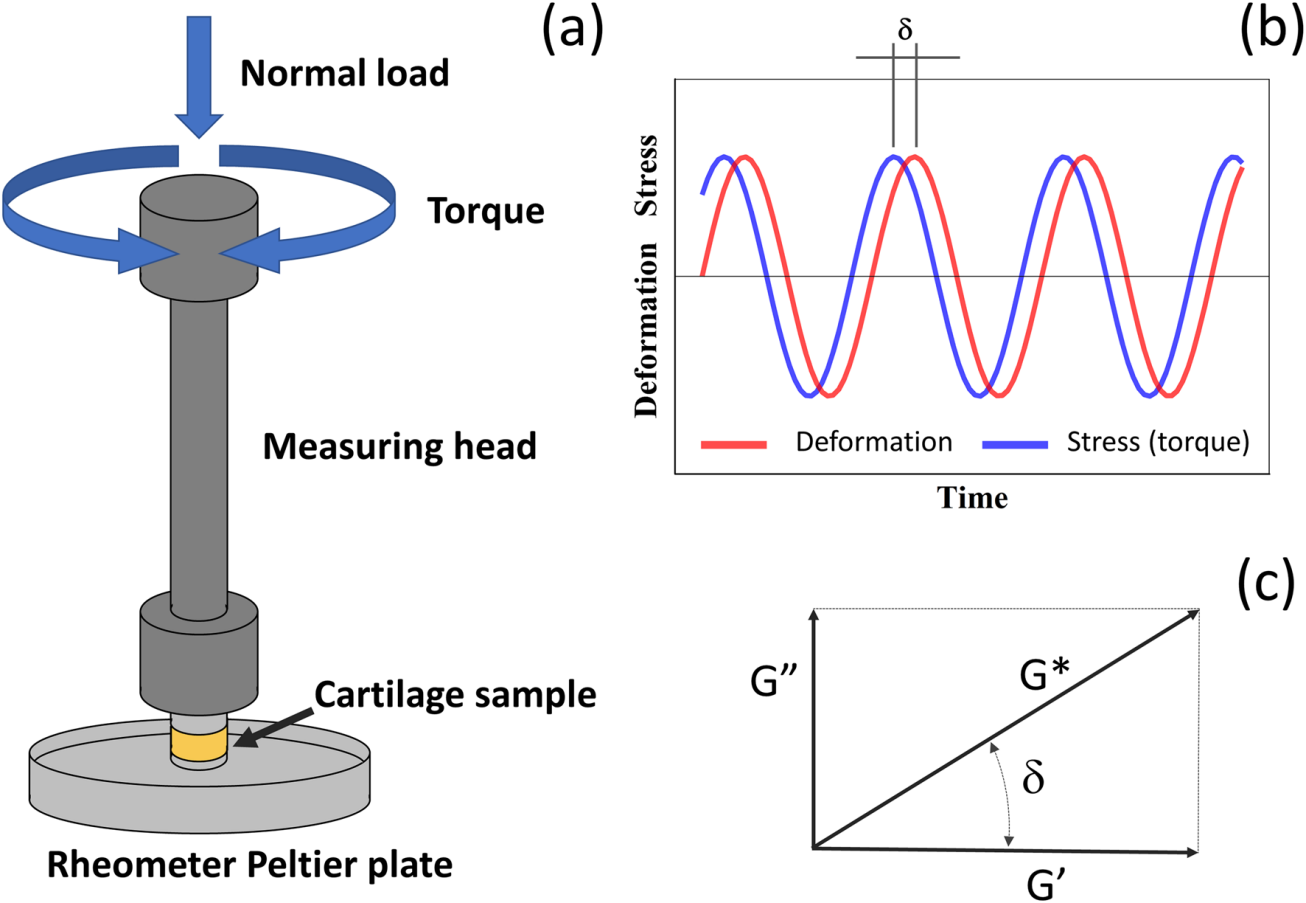
Scheme of (**a**) Peltier plates geometry used for the tests, (**b**) stress response to oscillatory strain deformation for a viscoelastic material and (**c**) relation between complex, elastic and loss moduli.

Experimental setup and data analysis The tests were performed using a rheometer (MRC702, Anton Paar Ltd, UK), equipped with purposely made 5 mm diameter circular flat plates. The top part component was equipped with a standard 5 mm diameter aluminium Peltier plate; the lower component of the measuring system was a Peltier plate made from aluminum; this was designed as a 5 cm diameter flat bottom plate with a raised center of 5 mm diameter (Fig. 2a).

Cartilage samples were carefully placed on the raise platform of the lower component then the top component was lowered until in contact with the cartilage sample (recorded applied force < 1 N); the reservoir represented by the lower component was filled with fresh PBS until the cartilage sample was immersed. 5 mm disks of a yellow rough paper were applied on top of cylindrical parts of plates using a medical adhesive in order to prevent sample sliding during experiments.

Normal forces were applied and the resultant pressure P (Pa) was calculated as follow:

1 $$ P\,\left( {{\text{Pa}}} \right) = { }\frac{{F\,\left( {\text{N}} \right)}}{{S\,\left( {{\text{m}}^{2} } \right)}} $$

where: F (N) is normal force applied by the rheometer, S (m^2^) is cartilage sample surface obtained by:

2 $$ S\,\left( {{\text{m}}^{2} } \right) = { }\frac{{\pi { } \times { }d^{2} }}{4} $$

where: d is the sample diameter.

During a typical experiment a sinusoidal deformation is applied to the top plate while the bottom plate remains stationary, imposing a time dependent strain on the sample (γ(t) = γ0sin(ωt)); at the same time the torque quantifies the stress applied

$$ \sigma = G^{\prime }\upgamma 0\sin \left( {\upomega {\text{t}}} \right) + {\text{iG}}^{\prime \prime }\upgamma 0\sin \left( {\upomega {\text{t}}} \right) $$

where γ0 is the strain when sin(ωt) is equal 1;

ω = 2p f, where f is the frequency of strain oscillation; t is time; i is the imaginary unit (Fig. 2b). Four physical values which describe the behaviour of articular cartilage linking strain and stress were collected during our tests. These are complex modulus G*, storage modulus G′, loss modulus G″ and phase angle δ (Fig. 2c); they are defined as follows:

3 $$ G^{*} = G^{\prime } + iG^{\prime \prime } $$

4 $$ \left| {G^{*} } \right| = \sqrt[2]{{\left| {G^{\prime } } \right|^{2} + \left| {G^{\prime \prime } } \right|^{2} }} $$

5 $$ \delta = \tan^{ - 1} \left( {\frac{{G^{\prime \prime } }}{{G^{\prime } }}} \right) $$

The complex modulus (G*) has a real and an imaginary part represented by G′ and G″, respectively. Storage modulus (G′) measures the deformation energy collected in the solid part of the cartilage, whereas loss modulus (G″) measures the dissipated energy from the fluid part; the phase angle reflects the angular offset between force and deformation.

The asymptotic nature of G* at high and low frequency allowed the definition of G^*^_low_ as the value of G^*^ exhibited by the tissues as $\mathop {\lim }\limits_{w \to 0} G^{*}$ and G^*^_high_ as the value of G^*^ exhibited by the tissues as $\mathop {\lim }\limits_{w \to \infty } G^{*}$^38–40^.

For environmental and test parameters, the Peltier plate temperature was fixed at 37 °C because it refers to human body temperature. The frequency sweep ranged from 0.001 to 10 Hz, the applied strain was set at 0.1% after initial tests and 10 points per decade were collected. In order to determinate strain to be used, a test was carried on an untreated sample, with an oscillation strain sweep ranging from 0.01% to 10% and at a fixed frequency of 1 Hz.

The frequency resulting in a local maximum of the phase angle maximum is defined as characteristic poroelastic relaxation frequency (*f*_*peak*_). Cartilage permeability was estimated from the following equation^39,40^:

6 $$ K_{H} = \frac{{f_{peak} \times d^{2} }}{{E_{L} }} $$

where: *K*_*H*_ hydraulic permeability, *f*_*peak*_ characteristic poroelastic relaxation frequency, *E*_*L*_ equilibrium modulus at low frequency, *d* characteristic contact distance, in our case *d*^*2*^ is the cartilage sample surface *S*.

After the experiment at a chosen applied load, samples were discarded (each sample undergo only a single sweep experiment); each sweep experiment was carried out on three samples obtained from three individual animals for a total of 9 samples.

## Results

**Figure 3:**
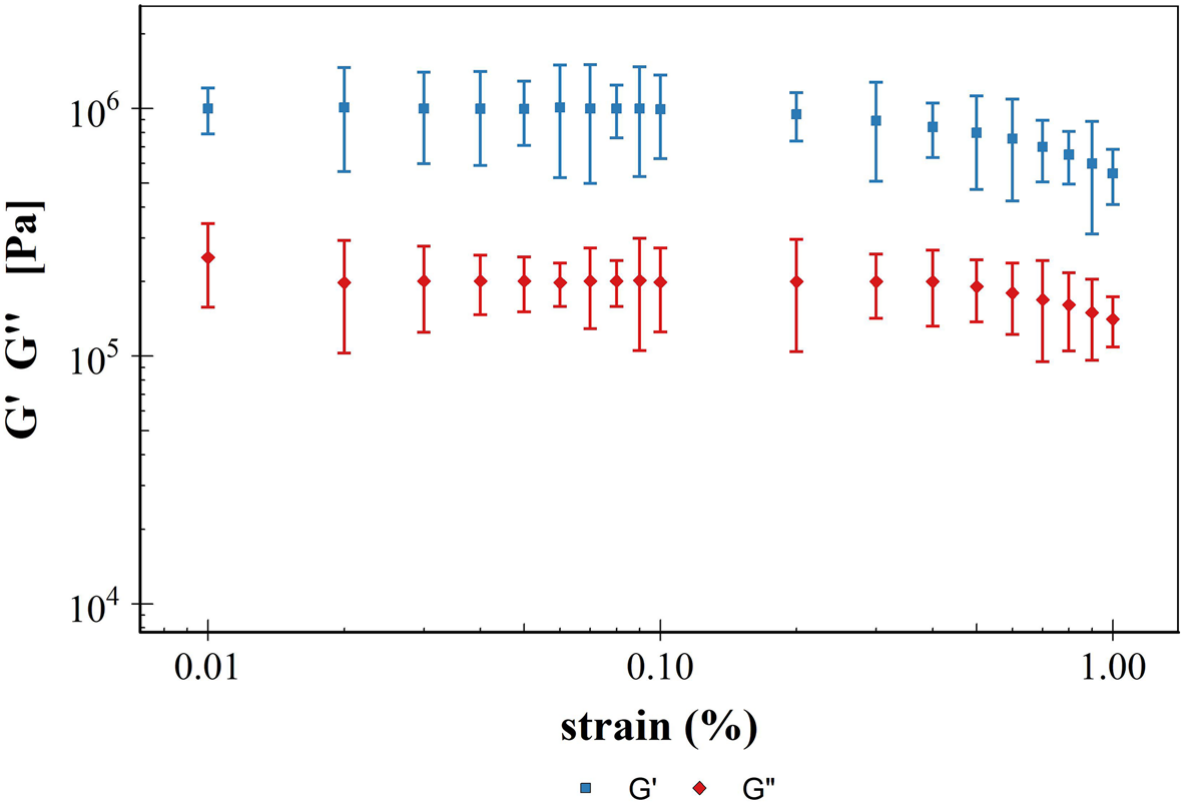
Storage modulus (G') and loss modulus (G″) of cartilage as function of applied strain at 1 Hz.

The dependence of the rheological properties of viscoelastic materials generally exhibits two regions; at low strain the storage and loss moduli are independent of strain while beyond this critical strain level these two moduli decline as the behaviour of the material is non-linear. Hence, the first operational parameter determined for the use of the rheometer for cartilage viscoelastic properties was the critical applied strain, this was carried out through a strain sweep test at a fixed frequency of 1 Hz. Storage modulus G′ and loss modulus G″ remained almost constant when the stain was in the range 0.01—0.1%; both moduli decreased with increasing strain when strains were greater than 0.1% (Fig. 3). Considering these results, all tests were then run at fixed 0.1% applied strain.

### Cartilage properties

**Figure 4:**
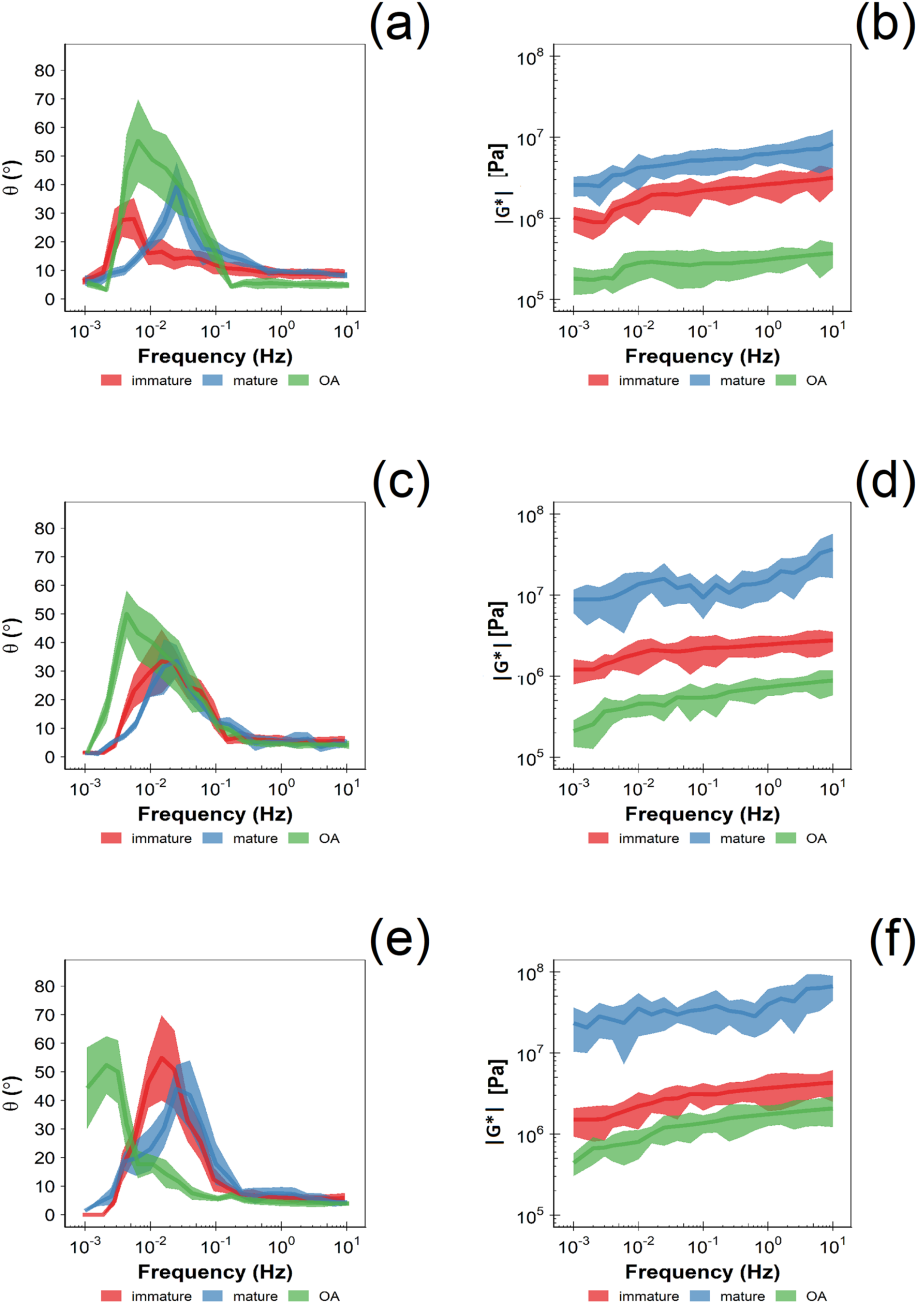
Dynamic shear modulus (right) and phase angle (left) of normal and GAG depleted cartilage samples at different frequencies under applied of load of 5 N (**a**, **b**), 25 N (**c**, **d**) and 50 N (**e**, **f**).

In all the three types of cartilage tissue tested (immature, mature and OA), both complex modulus G^*^ and phase angle δ were strongly dependent on both oscillatory frequency and applied load (Fig. 4). G^*^ monotonically increased with frequency with a sigmoidal pattern while δ exhibited a bell-shaped profile with regard to frequency.

In immature cartilage samples, G^*^ at the lowest frequency tested was about 1 MPa regardless of the applied load, while at 10 Hz it was about 3 MPa when the applied load was 5 and 25 N and 4.5 MPa for an applied load of 50 N. G^*^ of mature cartilage was greater than immature; at an applied load of 5 N it ranged from 2 MPa at 0.001 Hz to 6 Hz at 10 Hz; at 25 N and 50 N, G^*^ varied from 10 MPa at 0.001 Hz to 20 MPa at 10 Hz and from 16 MPa to30 MPa respectively. In GAG depleted cartilage samples, G^*^ at the lowest frequency tested was about 0.2 MPa when the applied load was 5 and 25 N and 0.4 MPa when the applied load was 50 N. At 10 Hz it was about 0.4, 0.9 and 2 MPa when the applied load was 5, 25 and 50 N, respectively.

The phase angle exhibited a bell shaped profile for all three cartilage types (immature, mature and GAG depleted) with values close to 0 at both 0.001 Hz and 10 Hz for all applied loads tested in immature and mature cartilage samples, while in GAG depleted cartilage the phase angle at 0.001 Hz increased up to 40° with increasing applied loads. The characteristic poro-elastic relaxation frequency (*f*_*peak*_), that is the frequency resulting in a local maximum of the phase angle, increased with increasing applied loads in immature cartilage; *f*_*peak*_ was 0.010 Hz when the applied load was 5 N; 0.016 Hz for an applied load of 25 N or 50 N. The opposite was observed in GAG-depleted cartilage as *f*_*peak*_ was 0.006 Hz when the applied load was 5 N, 0.004 Hz when the applied load was 25 N and 0.002 Hz at an applied load of 50 N. The characteristic poro-elastic relaxation frequency of mature cartilage was 0.025 Hz at all three applied loads tested.

**Figure 5:**
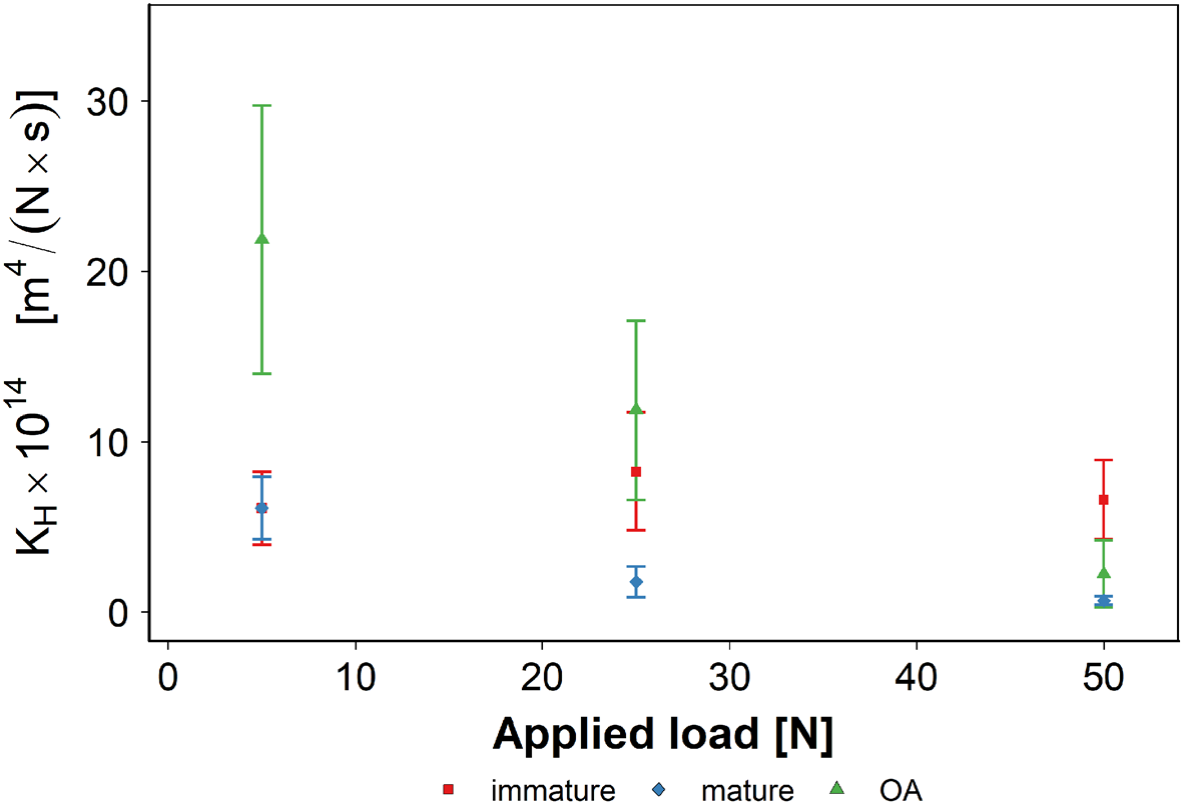
Hydraulic permeability (K_H_) of normal and GAG depleted cartilage at different applied loads.

For immature cartilage the hydraulic permeability (K_H_) did not appear to vary with the applied load and remained around 7 × 10^−14^ m^4^/ N; on the contrary, in mature and GAG depleted cartilage K_H_ decreased with increasing applied load (Fig. 5). At the lowest applied load use in this work (5 N) K_H_ of GAG depleted tissues was 21 × 10^−14^ m^4^/ N s while when the applied load was 50 N, K_H_ was 2 × 10^−14^ m^4^/ N. For mature cartilage K_H_ was 6 × 10^−14^ m^4^/ N s and 0.7 × 10^−14^ when the applied load was 5 and 50 N, respectively.

The difference between the K_H_ of immature and GAG depleted cartilage was statistically significant only at applied load of 5 N (p < 0.05). The difference between the K_H_ of mature and GAG depleted cartilage was statistically significant at applied load of 5 N and 25 N (p < 0.05). The difference between the K_H_ of mature and immature cartilage was statistically significant at applied load of 5 N and 25 N (p < 0.05).

**Figure 6:**
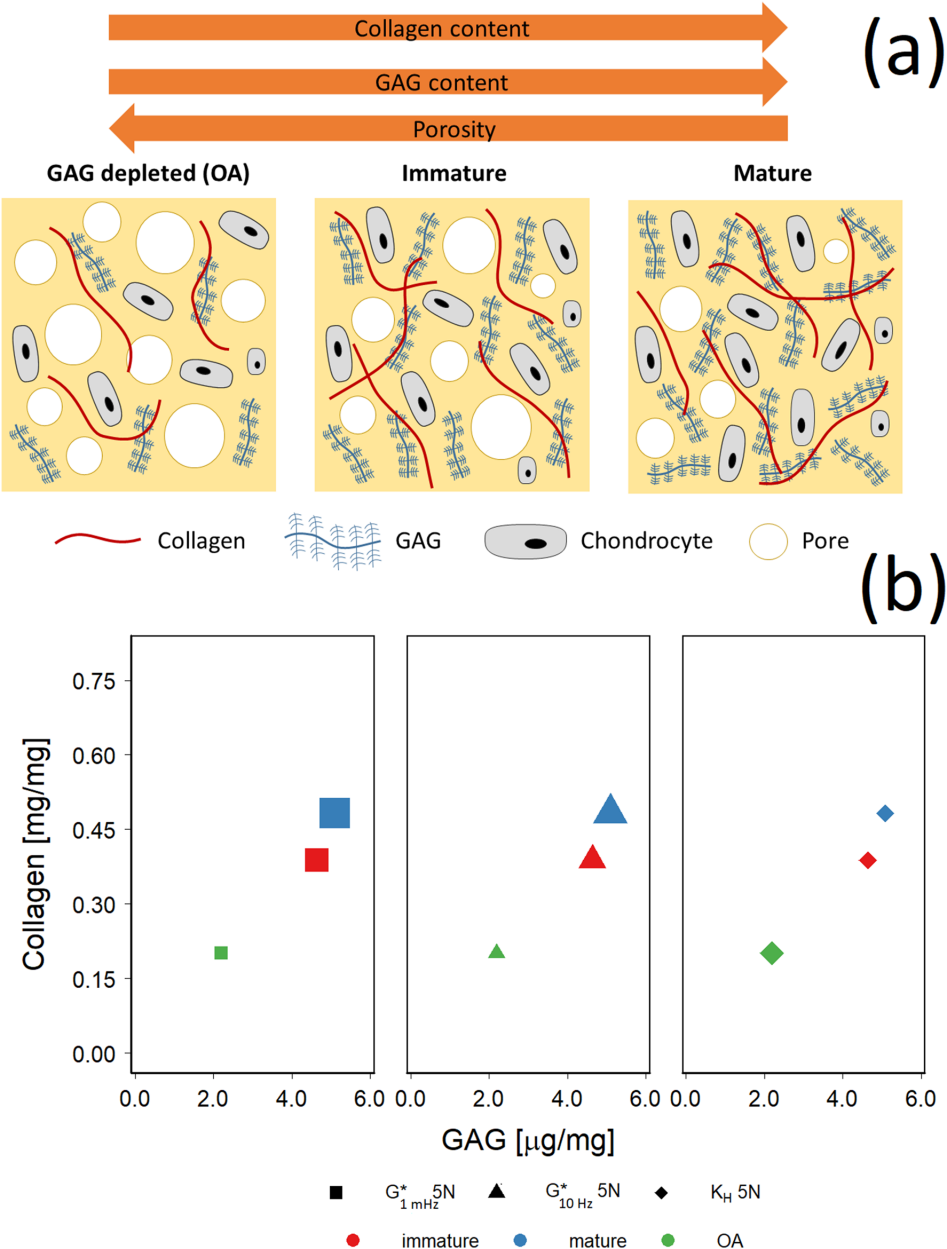
(**a**) Schematic representation of the structure/composition of different tissues used. (**b**) correlation among GAG, collagen and mechanical properties (G^*^_1 MHz,_ G^*^_10 Hz_ and hydraulic permeability (K_H_)) at 5 N applied load. Size of the symbols is proportional to the value of the parameter represented.

The amount of GAG and collagen in the different samples investigated appeared correlated (Fig. 6a), with the lowest amount of both molecules in GAG-depleted samples and the highest in mature samples. Moreover, the mechanical properties measured also appeared to be correlated with the amount of GAG/collagen (Fig. 6b). G^*^_1 MHz_ and G^*^_10 Hz_ increased with increasing GAG or collagen content (from GAG-depleted to immature and mature tissues); the opposite relation was observed for K_H_ that instead decreased with increasing GAG or collagen content.

## Discussion

The development of effective treatments for ailments affecting cartilage requires the ability to test samples with techniques capable of closely replicating biological stimuli observed in vivo coupled with ease of use and low capital and running costs. We have shown in this work that a commercial rheometer can be employed for testing the mechanical properties of healthy and GAG-depleted cartilage fulfilling such requirements. The applied loads in the experiments corresponded to pressures ranging between 0.2 and 2.5 MPa that is just under the maximum pressure, reported about 10 MPa, experienced by cartilage in joints during daily activities^41,42^; similarly, the frequency range employed in this study (0.001 to 100 Hz) represent typical daily activities; transient loading observed in physiological activities occurs at frequencies ranging from below 1 Hz for slow walking^43^ to around 100 Hz in the subgroup population with rapid heel-strike rise^44^.

The mechanical and physiological response of cartilage to stimuli depended on both the tissue composition and the spatial configuration of its components. For example, tissues stiffer then healthy sample are more prone to damage when subjected to high energy event^45^; also loss of ECM, and consequent reduction of GAG content, is progressively observed in cartilage affected by OA^46,47^. Analogously, during tissues maturation from childhood to adult the amount of GAG and collagens increase^48,49^. Cartilage mechanical properties are closely described by a poroelastic model where the material responses (e.g., stiffness, strength) depend on the frequency of the applied stimulus^50,51^, this behaviour is originated by the presence elastic component and porosity of the material and the flow of interstitial fluid through it. Poroelastic interactions regulate solute transport, energy dissipation, self-stiffening and lubrication thus are critical to biological and pathophysiological cartilage functions^52^. Moreover, under cyclic loading conditions, the applied stress and the resulting strain are not in phase^24^; this leads to the definition of the following quantities: storage modulus *E*^*’*^, loss modulus *E*^″^, dynamic elastic modulus E^*^ and phase angle (δ)^24^. When shear is considered instead of compression the analogous quantities are: G′, G″ and G^*^. The complexity exhibited by cartilage structure should be matched by testing methods capable of analysis resembling the in-vivo situations; simple methods such as GAG determination provide valuable evidence but can not capture detailed information about cartilage structure and properties. On the hand, material tests such as dynamic mechanical analysis (compression frequency sweep)^31–34^ and AFM nanoindentation^39,40,52,53^ can provide comprehensive knowledge regarding the poroelastic properties of cartilage; however they require considerable capital investments and highly skilled operators. There are also custom-made devices^62,63^ for confined-compression creep and stress relaxation tests exist for experiments on cartilage samples, which can be cheaper than currently available commercial rheometers^64^. In general, rheometers, conversely, are relatively inexpensive and easy to use devices; they have been employed in cartilage studies predominantly when configured as tribometer for the determination friction coefficients generally under conditions of constant load^35,54,55^. The values of G for bovine tissues, a good model for human cartilage^31^, obtained in this work are frequency depended and in the same region of those previously reported (0.2–2.0 MPa)^56^. Moreover, the observed increase in storage and loss moduli from GAG-depleted to, immature to mature is closely correlated to the amount of GAG and collagen in the ECM (Fig. 6); the lower shear rate measured in GAG depleted samples was similar to the observation of decreasing cartilage G′ with increasing OA grades^6^ or after proteoglycans degradation^57^.

A commercial rheometer had been utilised to determine cartilage frequency-dependent properties^58^ but did not observe a bell shape profile of the phase angle as function of the strain frequency and thus no K_H_ could be estimated. Such limited outcome is likely a consequence of the combination of the sample size (diameter 8 mm) and the applied load (1 N) employed in that study. Assuming the cartilage tissues had same K_H_ of those tested in this work, *f*_*peak*_ would have been at ~ 0.004 Hz with an applied load of 5 N, hence the lower load would have shifted the characteristic poroelastic relaxation frequency below the minimum frequency tested (0.001 Hz); the different tissues (lamb knee vs bovine ankle) could also results in different hydraulic permeabilities. These observations highlight how the experimental design and parameters choice must be carefully considered in order to maximise the information gathered from the experiments. Our choice of removing the underlying bone from the cartilage samples was informed by previous observations related to the role of subchondral bone on frequency dependent properties of cartilage^31–34^.

Cartilage parameters (G^*^ and K_H_) determined through the rheometer set-up presented in this work are in close agreement with those reported using more complex devices. For example hydraulic permeability of cartilage determined through AFM nanoindentation have been reported in the range 10^−14^ m^4^/N s^38,40,59^ with GAG depleted tissues exhibiting reduced resistance to interstitial fluid flow (higher values of K_H_) than the corresponding normal tissues^38,39,53^ in consequence of the increased porosity resulting from the degradation of aggrecan^60^; the opposite (lower K_H_ in light of reduced porosity) was observed in mature sample that are known to increase GAG and collagen content during the transition from childhood to adulthood^49^. Similarly, the decrease of hydraulic permeability observed with increasing applied load can be attributed to the compression of the pore^61^.

Although our data are in good agreement with data from the literature that were obtained by different methodologies, we must emphasise that our study was conducted under an assumption that cartilage is an isotropic material. This is one of the limitations of our approach. Moreover, despite the permeability in this study (Fig. 5) is consistent with some of the previous results, there are other reports showing that permeability is an order of magnitude lower; i.e., order of 10^−15^ m^4^/N s^61,62,64^.

Another limitation is that only radial permeability was measured in this study since the methodology did not allow measurements of the axial permeability. While data from the literature showed that for compressions > 10% strain, the axial permeability was significantly greater, by a factor of 5–10, than the radial permeability, whereas for compression < 10% strain there were no significant differences^64^. The latter corresponded to the strain range used in our study, therefore, we based our approach on this information from the literature.

## Conclusions

Understanding of the fundamental biological mechanisms involved in cartilage metabolism and the design of effective therapies or engineering tissues to treat diseases such as osteoarthritis require the investigation of the frequency-dependent viscoelastic properties of such tissue. These investigations can be currently carried out with complicated, expensive and difficult to operate equipment thus alternatives are needed.

We have developed a methodology employing a commercial rheometer for the determination of frequency-dependent viscoelastic properties of cartilage that are critical for their functionality and affected by pathologies. This device is relatively inexpensive and easy to operate, nevertheless the parameters measured matched those obtained in more complex testing. Therefore, such methodology could accelerate the development and validation of the efficacy and efficiency of treatments aimed at replacing damaged cartilage, for example comparing healthy tissues with engineered samples. Moreover, the device could also provide a diagnostic tool as the different poroelastic properties of healthy and OA tissues.
